# Energy Conservation and Production Efficiency Enhancement in Herbal Medicine Extraction: Self-Adaptive Decision-Making Boiling Judgment via Acoustic Emission Technology

**DOI:** 10.3390/ph18101556

**Published:** 2025-10-16

**Authors:** Jing Lan, Hao Fu, Haibin Qu, Xingchu Gong

**Affiliations:** 1 College of Pharmaceutical Sciences, Zhejiang University, Hangzhou 310058, China; 12319005@zju.edu.cn (J.L.); 12119016@zju.edu.cn (H.F.); quhb@zju.edu.cn (H.Q.); 2 State Key Laboratory of Chinese Medicine Modernization, Hangzhou 310058, China; 3 Jinhua Institute of Zhejiang University, Jinhua 321016, China

**Keywords:** extraction process, boiling judgment, acoustic emission, self-adaptive modeling, self-decision judgment

## Abstract

**Background:** Accurately detecting the onset of saturated boiling in herbal medicine extraction processes is critical for improving production efficiency and reducing energy consumption. However, the traditional monitoring methods based on temperature suffer from time delays. To address the challenge, acoustic emission (AE) signals were used in this study owing to its sensitivity to bubble behavior. **Methods:** An AE signal acquisition system was constructed for herbal extraction monitoring. Characteristics of AE signals at different boiling stages were analyzed in pure water systems with and without herbs. The performance of AE-based and temperature-based recognition of boiling stages was compared. To enhance applicability in different herb extraction systems, multivariate statistical analysis was adopted to compress spectral–frequency information into Hotelling’s T^2^ and SPE statistics. For real-time monitoring, a self-adaptive decision-making boiling judgment method (BoilStart) was proposed. To evaluate the robustness, the performance of BoilStart under different conditions was investigated, including extraction system mass and heating medium temperature. Furthermore, BoilStart was applied to a lab-scale extraction process of Dabuyin Wan, which is a practical formulation, to assess its performance in energy conservation and efficiency improvement. **Results:** AE signal in the 75–100 kHz frequency band could reflect the boiling states of herbal medicine extraction. It was more sensitive to the onset of saturated boiling than the temperature signal. Compared with SPE, Hotelling’s T^2^ was identified as the optimal indicator with higher accuracy. BoilStart could adaptively monitor saturated boiling across diverse herbal systems. The absolute error of BoilStart’s boiling determination ranged from 1.5 min to 2.0 min. The increasing-temperature time was reduced by about 22–36%. For the extraction process of Dabuyin Wan, after adopting BoilStart, the increasing-temperature time was reduced by about 29%, and the corresponding energy consumption was lowered by about 26%. **Conclusions:** The first AE-based method for precise boiling state detection in herbal extraction was established. BoilStart’s model-free adaptability met industrial demands for multi-herb compatibility. This offered a practical solution to shorten ineffective heating phases and reduce energy consumption.

## 1. Introduction

Solid–liquid extraction is one of the core processes in the production of herbal medicines. The process control level directly affects the final product quality and production energy efficiency. Water decoction is most commonly used in herbal medicine production. Extraction time is usually counted from the onset of boiling. In the industry, the temperature sensor inside the extraction tank is utilized to identify the onset of boiling. However, temperature measurements often suffer from significant lag. When the system temperature reaches 100 °C, boiling is often vigorous, leading to unnecessary energy consumption [1]. Visual inspection is sometimes used to assist in judging boiling. It is prone to disturbances by tank lighting and herbal foam occlusion. It also has significant subjective bias. Ultimately, this results in longer increasing-temperature time and higher energy consumption. Therefore, in order to improve production efficiency and reduce energy consumption, there is an urgent need to develop an accurate and reliable online analysis method for boiling states.

Acoustic emission (AE) is a non-invasive process detection technique [2,3,4,5,6]. It can be used to sense transient elastic waves generated in industrial systems during rapid energy release [7,8,9,10]. The various stages of bubble nucleation, growth, detachment, oscillation, and collapse excite acoustic signals. These signals appear in specific frequency bands [11,12,13]. The bubble behavioral dynamics correspond to the different boiling stages (subcooled boiling, saturated boiling, etc.) [14,15,16]. Therefore, the spectral features of the AE signals can be used for identifying the boiling state. Zhang et al. [17] developed a real-time detection method based on correlations between AE signal power and heat flux to predict boiling status in flowing liquids. Seok et al. [18] validated the feasibility of AE in distinguishing boiling phases. However, existing studies have mainly focused on homogeneous liquid phase systems [19], and no application of AE technology for boiling judgment in solid–liquid mixtures during the herbal decoction process has been reported. Univariate and multivariate monitoring methods are mostly used in the field of boiling judgment with AE signal. AE features extracted by time domain analysis or frequency domain analysis are commonly used in method development [20,21,22]. Machine-learning and deep-learning models are also used to learn discriminative features for boiling state classification [23,24,25,26]. However, these approaches often require a prebuilt feature library or offline training, which limit their universality. AE spectra are influenced by factors including heating wall material characteristics [27,28,29], heating surface geometry [30,31,32], and boiling system composition [33,34,35]. There are many types of herbal medicines extracted by water decoction. Thus, a robust and accurate AE-based boiling judgment method universally applicable to diverse herbal systems is urgently needed.

In this study, AE technology was used to monitor boiling behavior during the extraction process of herbal medicine. Firstly, an AE signal acquisition setup for the extraction process was constructed. The correspondence between AE signals and boiling stage was established by analyzing the changes in AE signal and the behavior of boiling bubbles during the boiling process. On this basis, the spectral characteristics of AE signals under different extraction conditions and their feasibility of monitoring the boiling state were investigated. Then, a self-adaptive decision-making boiling judgment method (BoilStart) based on iterative multivariate statistical analysis was proposed. BoilStart was used for online monitoring of boiling state under different extraction conditions. Finally, BoilStart was applied to the extraction of Dabuyin Wan. The online judgment ability of the boiling state and the level of energy conservation and efficiency improvement were verified.

## 2. Results

### 2.1. Relationship Between AE Signal and Boiling Behavior in Pure Water

In this study, pure water was first selected as the test medium for cold-model experiments. The water was gradually heated until boiling occurred. A smartphone was used to record images, and AE signals were acquired during the process. Based on the observed bubble behavior, the boiling process was divided into two stages: subcooled boiling stage and saturated boiling stage.

**Figure 1:**
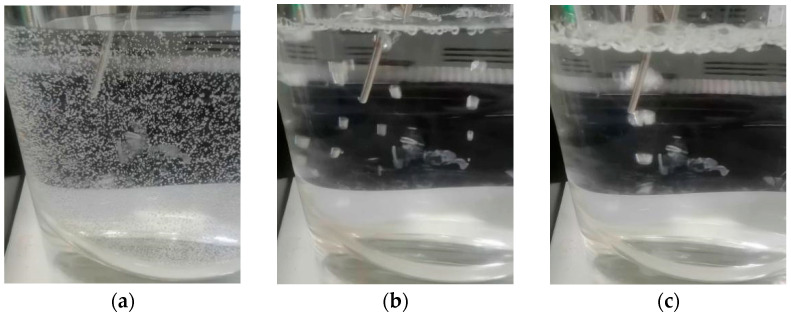
Behavior of bubbles at different boiling stages: (**a**) subcooled boiling; (**b**) discrete saturated boiling; (**c**) polymerized saturated boiling.

Figure 1a corresponds to the subcooled boiling stage. When the heating surface temperature approached the water saturated temperature, microbubbles formed slowly on the wall surface. No bubbles escaped from the water surface at this stage. As shown in Figure 1b, when the system entered saturated boiling, the bubbles detached from the wall surface, rose to the liquid surface, and eventually burst. This state, in which bubbles remained separated from each other, was referred to in this paper as “discrete saturated boiling”. When the boiling was more intense, the bubbles may merge with each other, as shown in Figure 1c. In this paper, this situation was referred to as “polymerized saturated boiling”.

**Figure 2:**
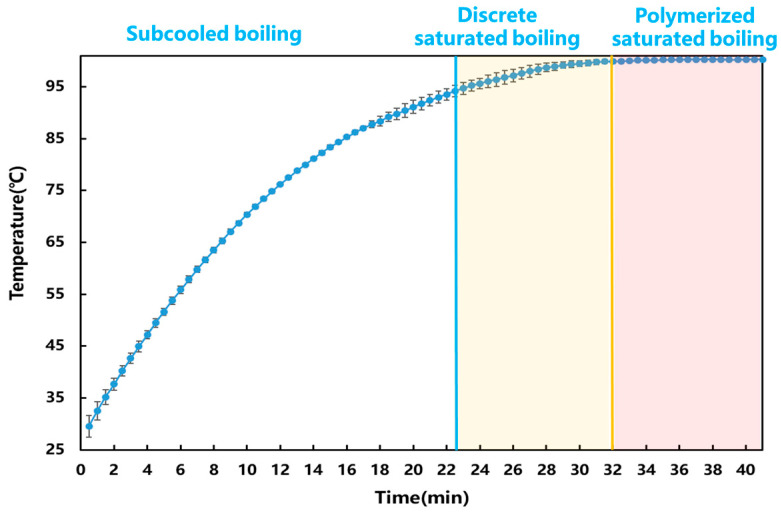
Changes in temperature at different boiling stages. (The blue vertical line indicates that the system changed from subcooled boiling to discrete saturated boiling, and the yellow vertical line indicates that the system changed from discrete saturated boiling to polymerized saturated boiling.)

Figure 2 shows the corresponding temperatures at different boiling stages. The temperature changes in the system at different boiling stages were not significant. Therefore, it was difficult to rely on temperature to accurately determine the stages.

**Figure 3:**
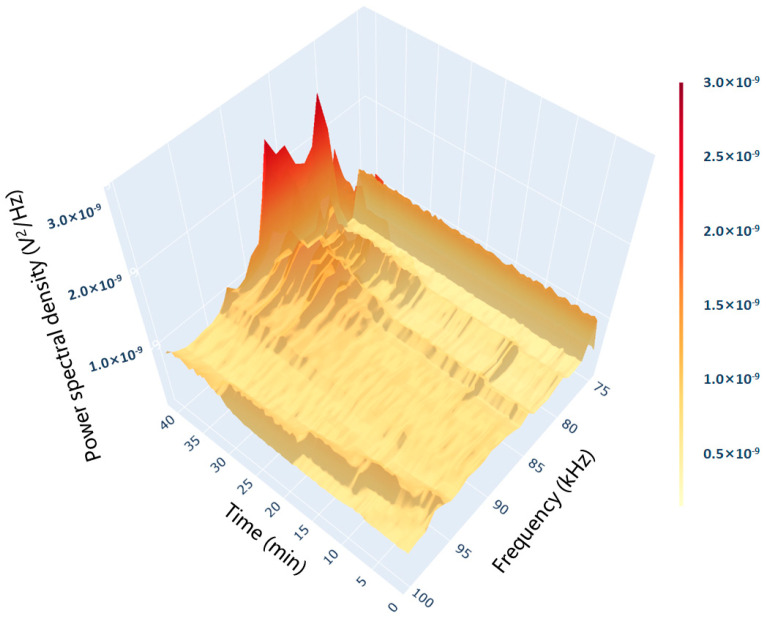
Characteristic spectrum of AE signal during the boiling process. (Both the axes and the color bars were linear. The power spectral density was presented in linear units (V^2^/Hz) and displayed in scientific notation.)

Observation of the spectrum in Figure 3 shows the presence of signal peaks in the 75–100 kHz band. In this study, the formation of microbubbles on the heating wall surface was taken as a sign that the system entered subcooled boiling. The rise of bubbles to the liquid surface after detaching from the wall surface was taken as a sign that the system entered discrete saturated boiling. The emergence of gas columns merging with each other was taken as a sign that the system entered polymerized saturated boiling. The boiling process was divided into three stages based on these indicators.

**Figure 4:**
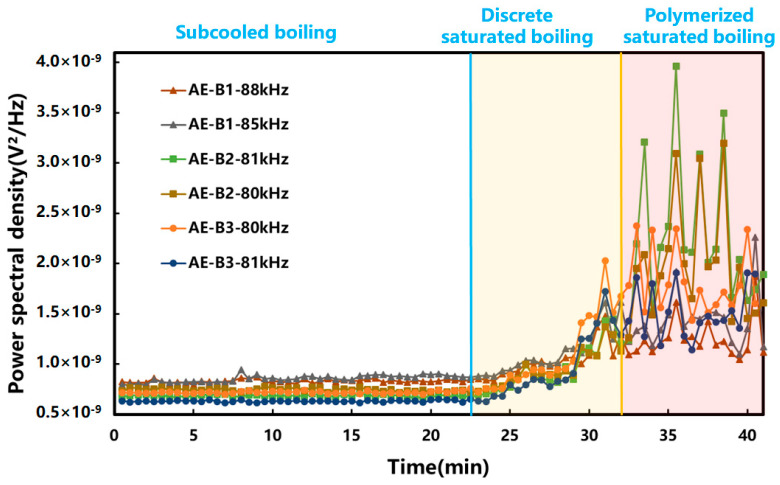
Variation in power spectral density under different boiling stages. (The blue vertical line indicates that the system changed from subcooled boiling to discrete saturated boiling, and the yellow vertical line indicates that the system changed from discrete saturated boiling to polymerized saturated boiling. Both the axes and the color bars were linear. The power spectral density was presented in linear units (V^2^/Hz) and displayed in scientific notation.)

In order to study the AE signal changes under different boiling stages more intuitively, the frequencies whose power spectral density trends matched different boiling stages were preferred. As shown in Figure 4, the amplitude of the AE signal did not change much in the subcooled boiling stage. In the discrete saturated boiling stage, the high-frequency detachment of bubbles caused violent oscillations in the water body, so the amplitude of the AE signal increased rapidly. Entering the polymerized saturated boiling stage, the inter-bubble interactions inhibited the fluid motion near the heating wall, and the oscillatory impact of the water body on the wall was reduced. As a result, the intensity of the AE signal decreased. Overall, the AE technique can reflect the different stages of the boiling process by its trends.

### 2.2. Stage-Wise Correlation Analysis Between AE Signals and Boiling Behavior During the Aqueous Extraction of Different Herbs

#### 2.2.1. Characterization of Boiling by “Signature Frequency”

**Figure 5:**
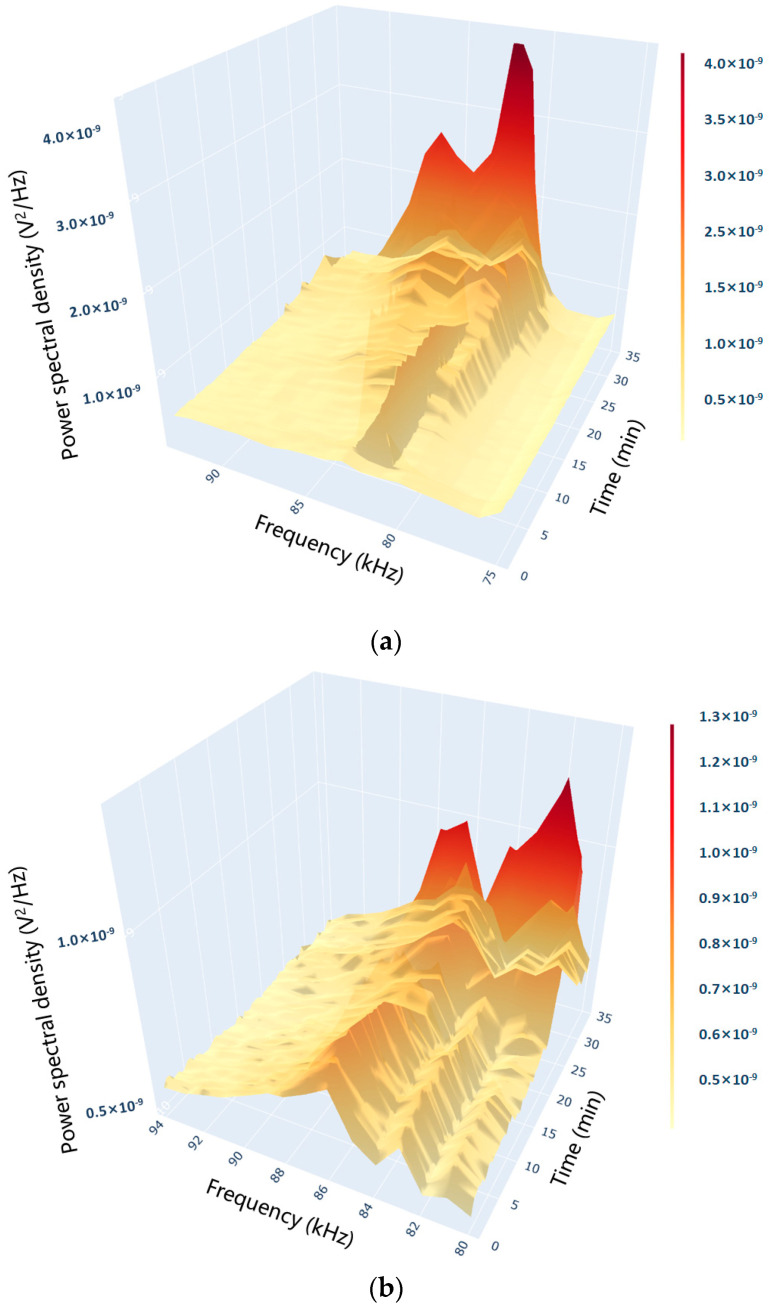

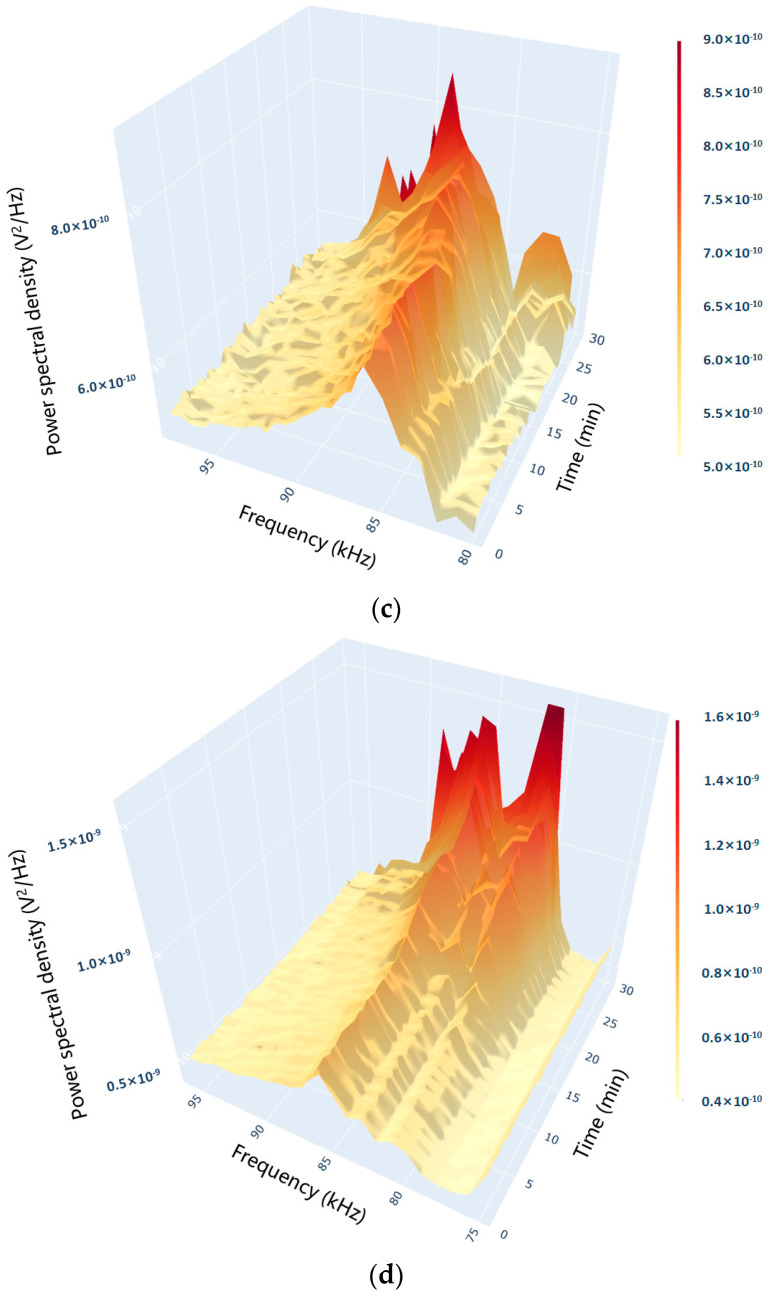
Characteristic spectra of different herbal medicine during the boiling process: (**a**) Rehmanniae Radix Preparata; (**b**) Phellodendri Chinensis Cortex; (**c**) Anemarrhenae Rhizoma; (**d**) Prunellae Spica. (Both the axes and the color bars were linear. The power spectral density was presented in linear units (V^2^/Hz) and displayed in scientific notation.)

To investigate the acoustic characteristics of different kinds of herbs, AE signals of the aqueous extraction systems of Radix Rehmanniae Praeparata, Phellodendri Chinensis Cortex, Anemarrhenae Rhizoma, and Prunellae Spica were collected as examples. The results are shown in Figure 5. The characteristics of the AE signals of the different systems were different. However, the overall trends were consistent with those of the pure water system described in Section 2.1.

**Figure 6:**
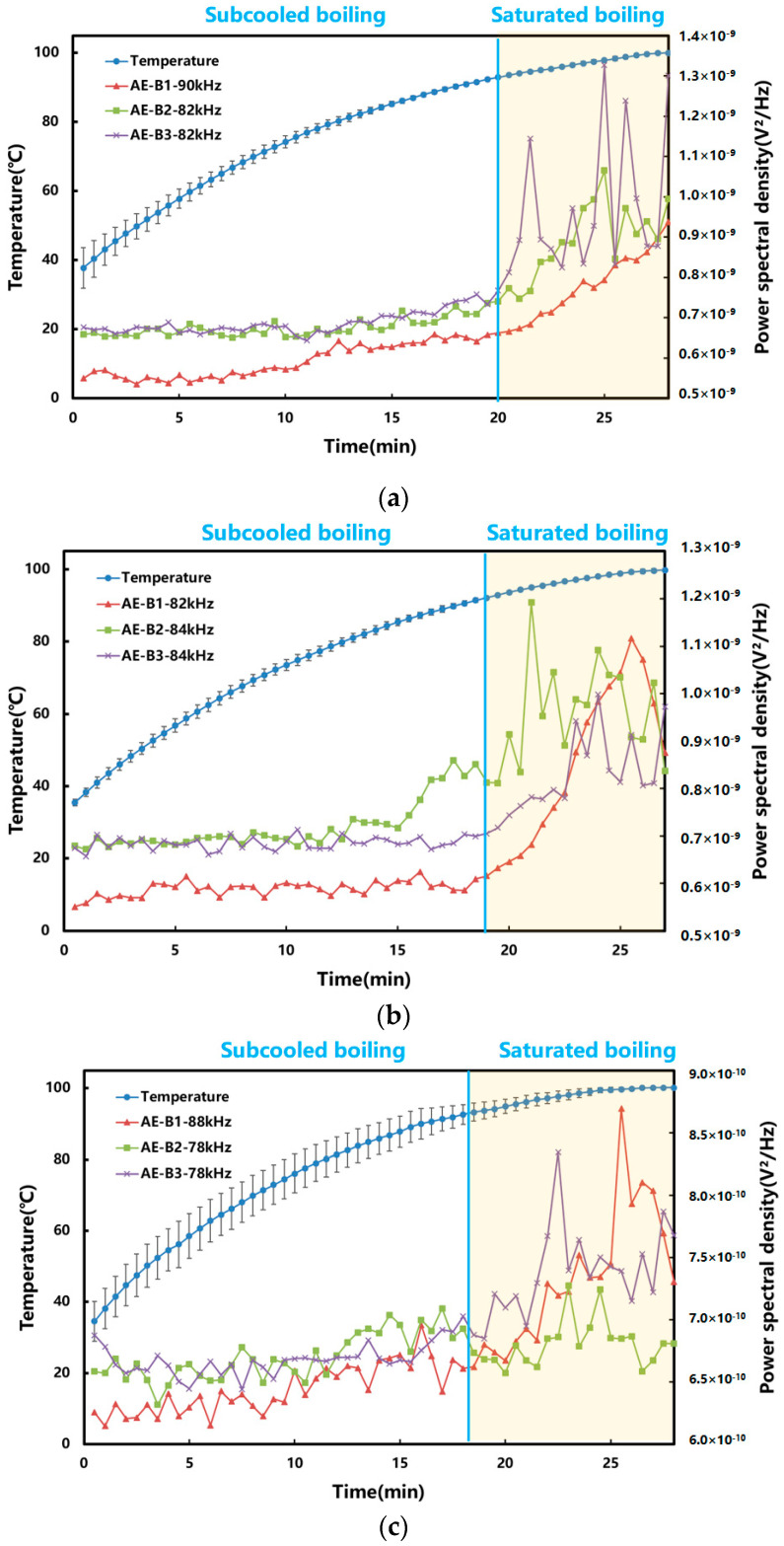

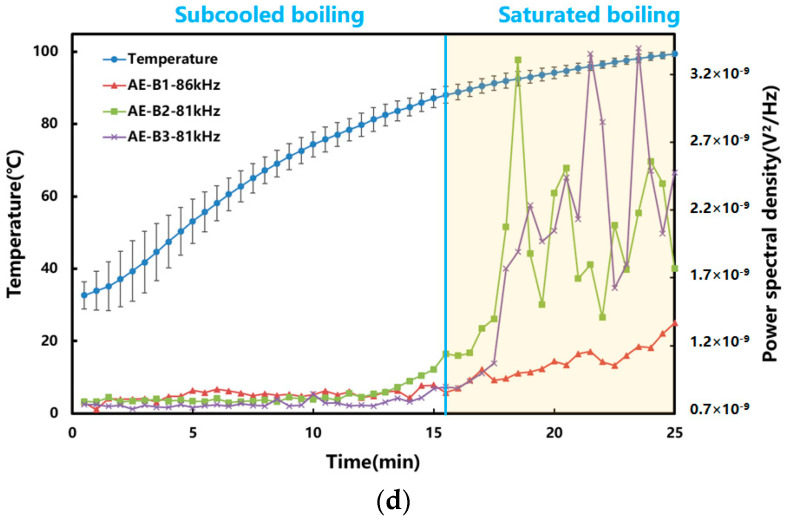
The variation trend of the increasing-temperature curve and the power spectral density at the “signature frequency” during the water extraction process of different herbal medicine. (The blue vertical line indicates that the system entered saturated boiling. Both the axes and the color bars were linear. The power spectral density was presented in linear units (V^2^/Hz) and displayed in scientific notation.) (**a**) Rehmanniae Radix Preparata; (**b**) Phellodendri Chinensis Cortex; (**c**) Anemarrhenae Rhizoma; (**d**) Prunellae Spica.

The rising of the bubbles to the liquid surface after detachment from the wall was regarded as a sign that the system entered saturated boiling. The “signature frequencies” that could reflect different boiling stages were manually identified. The results are shown in Figure 6. The “signature frequencies” varied in different herbal extraction systems. The temperatures at the onset of saturated boiling for Radix Rehmanniae Praeparata, Phellodendri Chinensis Cortex, Anemarrhenae Rhizoma, and Prunellae Spica were 92.5 ± 0.5 °C, 91.9 ± 0.8 °C, 92.8 ± 2.2 °C, and 88.5 ± 2.7 °C, respectively. Table 1 demonstrates the increasing-temperature time required by both methods. In the industry, reaching 100 °C is generally regarded as the onset of boiling. Compared with the industrial method, the AE-based method can shorten the increasing-temperature time of the four extraction systems of Radix Rehmanniae Praeparata, Phellodendri Chinensis Cortex, Anemarrhenae Rhizoma, and Prunellae Spica by 28.8 ± 3.9%, 30.5 ± 5.0%, 28.9 ± 1.6%, and 38.7 ± 1.8%, respectively. This suggested that the use of AE technology shortened the total duration of the extraction process, which in turn reduced the energy consumption.

#### 2.2.2. Characterization of Boiling by Multivariate Statistical Analysis

**Figure 7:**
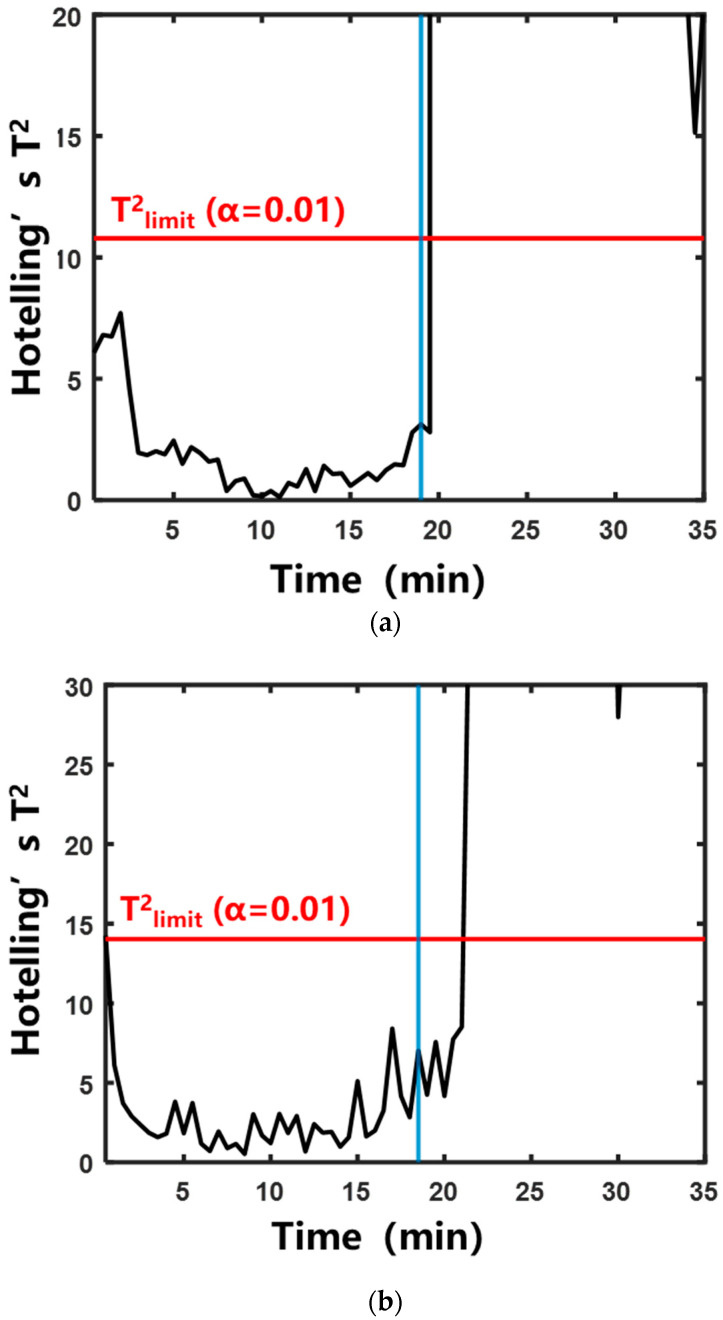

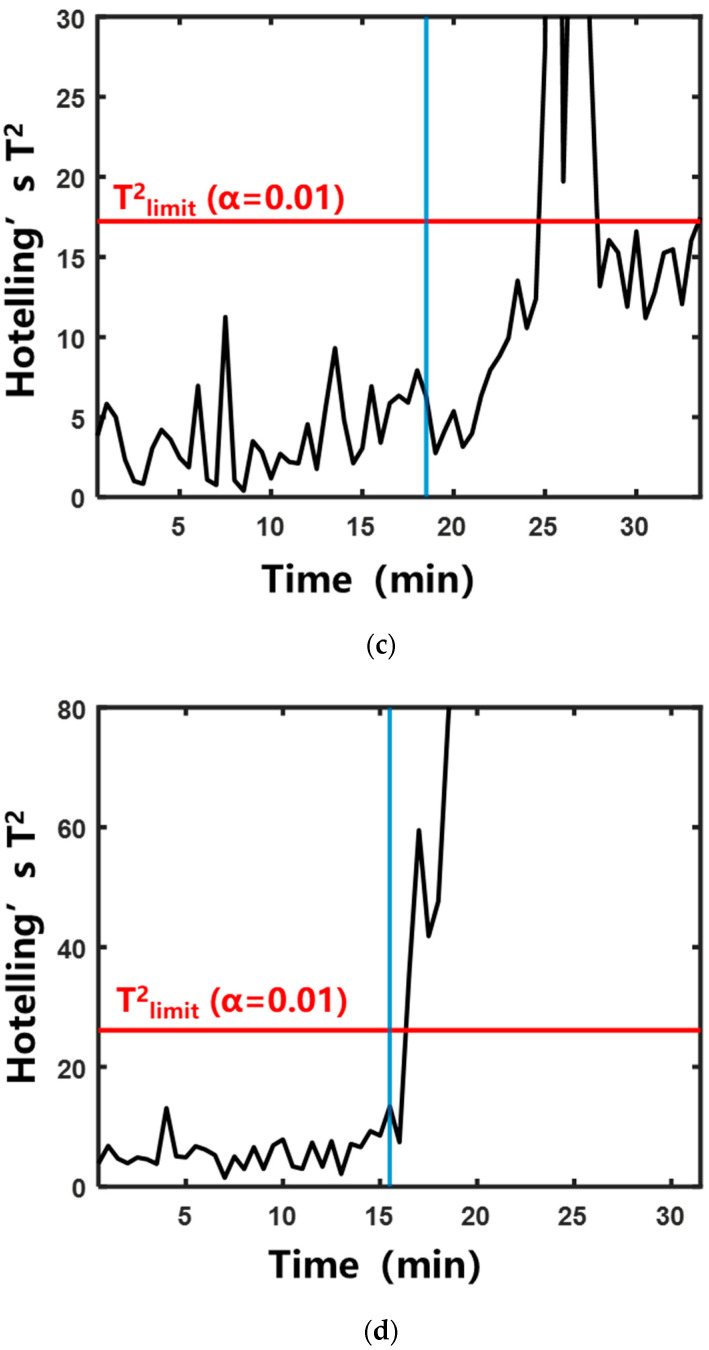
Control chart of Hotelling’s T^2^ statistic during the aqueous extraction of different herbs. The blue vertical line indicates that the system entered saturated boiling: (**a**) Rehmanniae Radix Preparata; (**b**) Phellodendri Chinensis Cortex; (**c**) Anemarrhenae Rhizoma; (**d**) Prunellae Spica.

**Figure 8:**
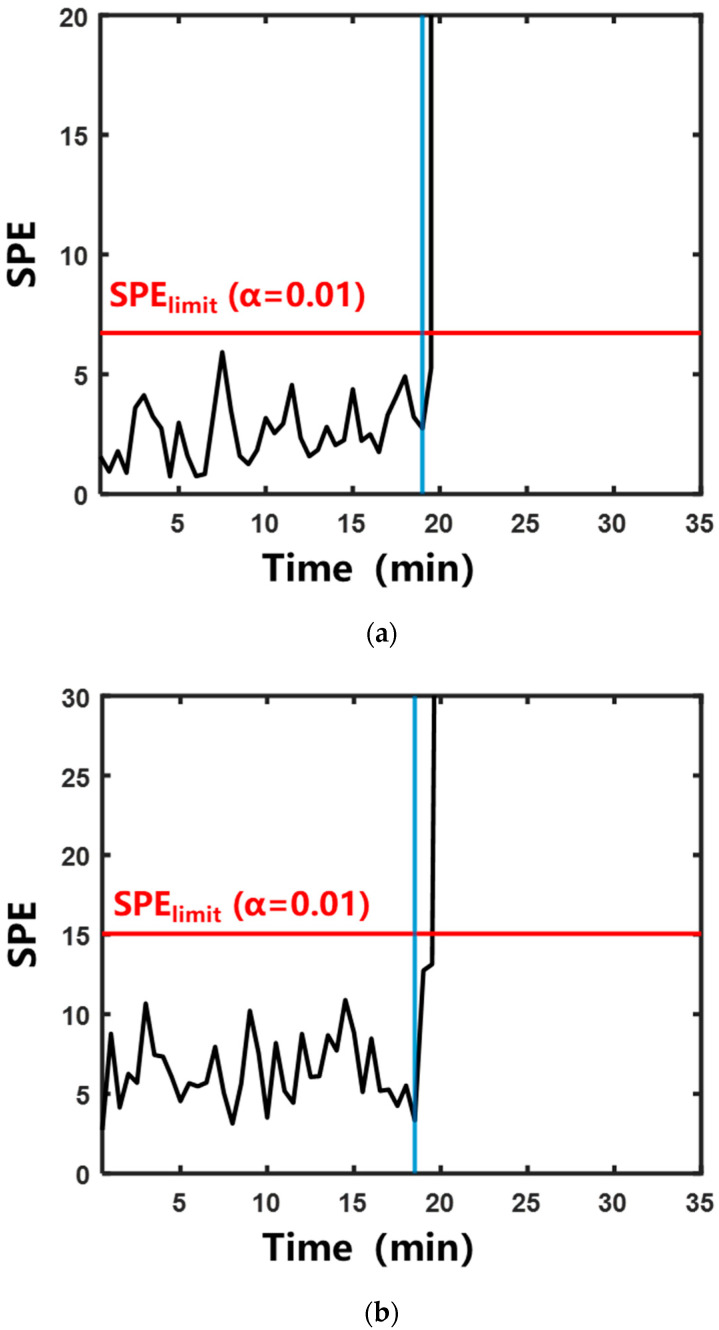

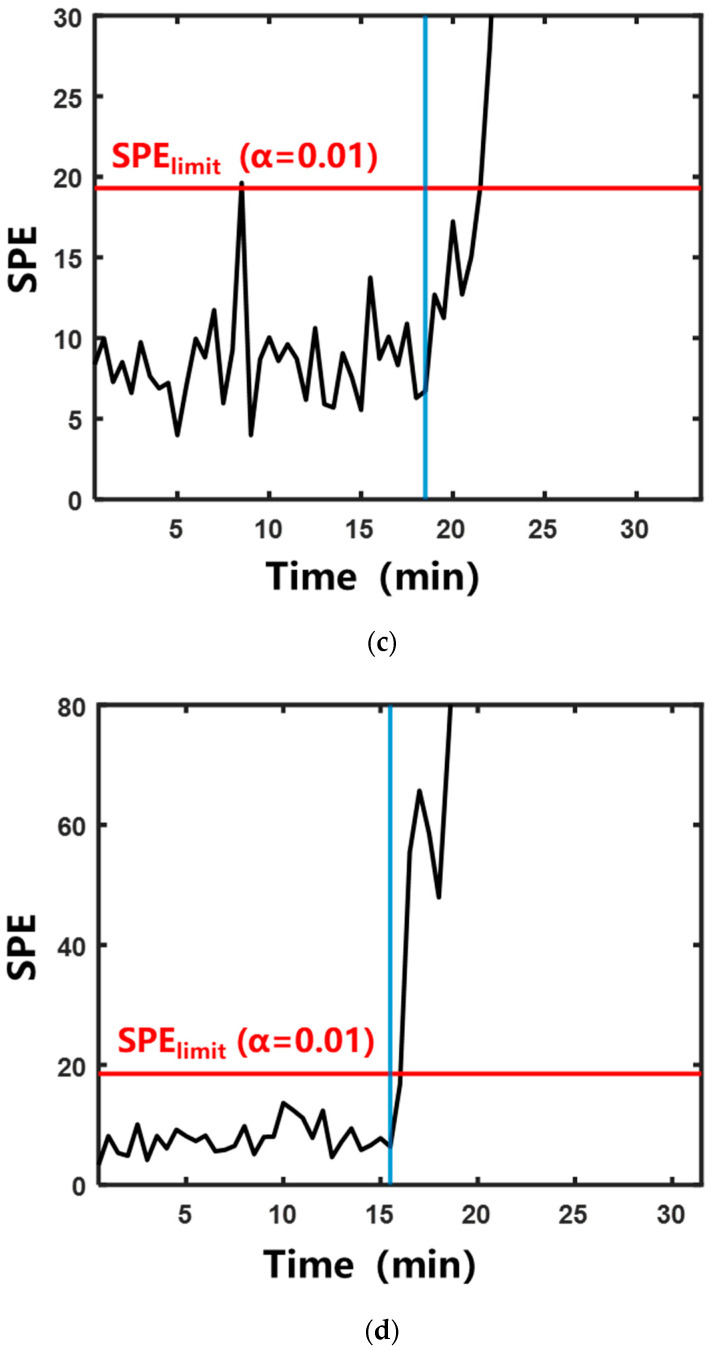
Control chart of SPE statistics during aqueous extraction of different herbs. The blue vertical line indicates that the system entered saturated boiling: (**a**) Rehmanniae Radix Preparata; (**b**) Phellodendri Chinensis Cortex; (**c**) Anemarrhenae Rhizoma; (**d**) Prunellae Spica.

To address the variation in “signature frequencies” across different herbal extraction systems, the multivariate statistical analysis method was adopted. This method eliminated the need for manual screening of “signature frequencies”. The specific calculation method is described in Section 4.4.2. Figure 7 and Figure 8 show the control charts of the two statistics during the aqueous extraction of different herbs. The specific moments exceeding the control limits are shown in Table 1. In the systems of Rehmanniae Radix Preparata, Phellodendri Chinensis Cortex, and Prunellae Spica, Hotelling’s T^2^ statistic and SPE statistic increased rapidly and exceeded the control limits after entering the saturated boiling state. In the Anemarrhenae Rhizom system, the SPE statistic exceeded the control limit near the onset of the subcooled boiling. Hotelling’s T^2^ statistic exceeded the control limit at saturated boiling. This suggests that the process trajectory of Hotelling’s T^2^ statistic can distinguish the subcooled and saturated boiling stages more accurately than the SPE statistic.

#### 2.2.3. Characterization of Boiling by BoilStart

**Figure 9:**
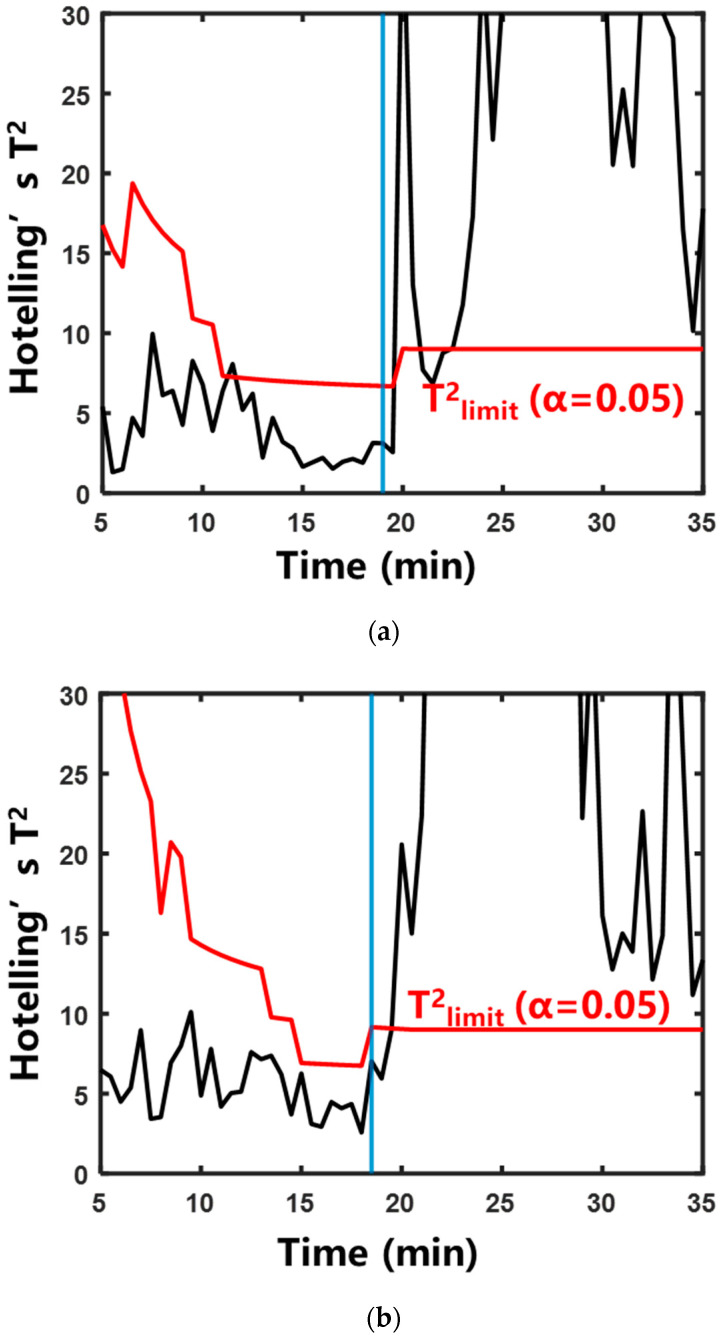

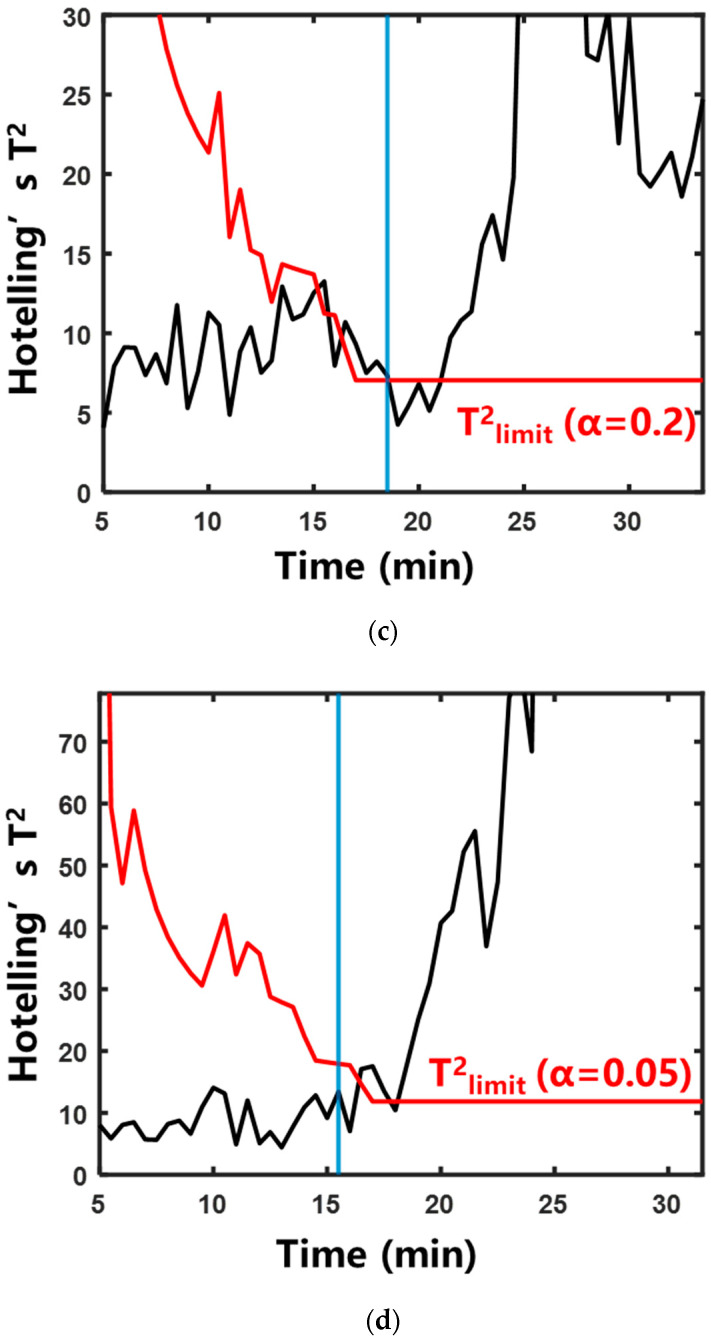
Evaluation of saturated boiling onset timing determined by BoilStart under varying extraction herbal medicine. The blue vertical line indicates that the system entered saturated boiling: (**a**) Rehmanniae Radix Preparata; (**b**) Phellodendri Chinensis Cortex; (**c**) Anemarrhenae Rhizoma; (**d**) Prunellae Spica.

BoilStart is a self-adaptive decision-making boiling judgment method based on iterative multivariate statistical analysis. BoilStart was used for the boiling start judgment of the aqueous decoction process of single herbs. The experimental results are shown in Figure 9 and Table 2. In different herb systems, the absolute errors were 1.8 ± 0.6, 2.0 ± 0.5, 1.8 ± 0.8, and 1.8 ± 0.6 min, respectively. The trigger was defined as Hotelling’s T^2^ statistic exceeding the control limit at two consecutive time points. The reference was the system’s entry into saturated boiling. This indicated that the prediction results of BoilStart were relatively accurate. Compared with the temperature method, BoilStart reduced the increasing-temperature time of the four extraction systems by 22.2 ± 5.2%, 23.1 ± 5.5%, 36.2 ± 2.7%, and 31.6 ± 0.3%, respectively. This indicated that BoilStart can improve efficiency.

### 2.3. BoilStart Predictive Capability Evaluation

The boiling state during the aqueous extraction was affected by a combination of factors such as the temperature of heating medium and total mass of the extraction system. In order to further evaluate the ability of BoilStart, it was used under different heating medium temperatures and extraction system masses.

#### 2.3.1. Extraction System Mass

**Figure 10:**
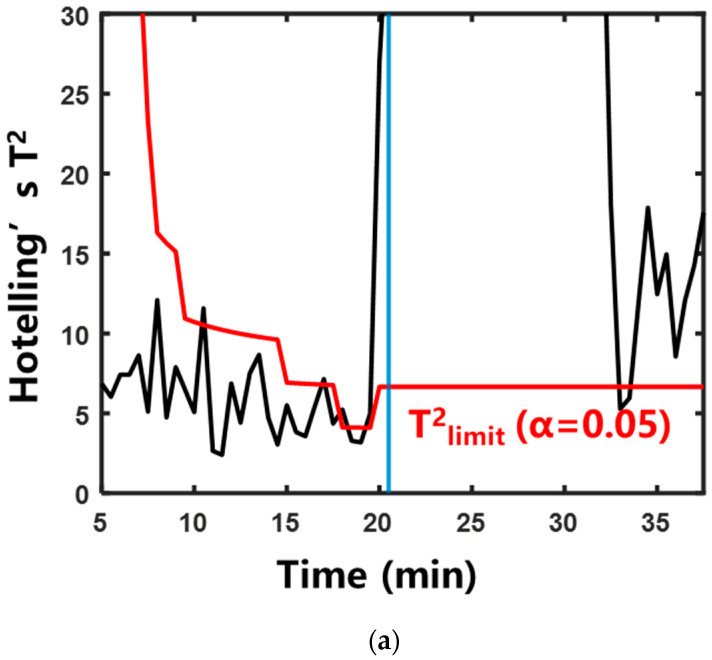

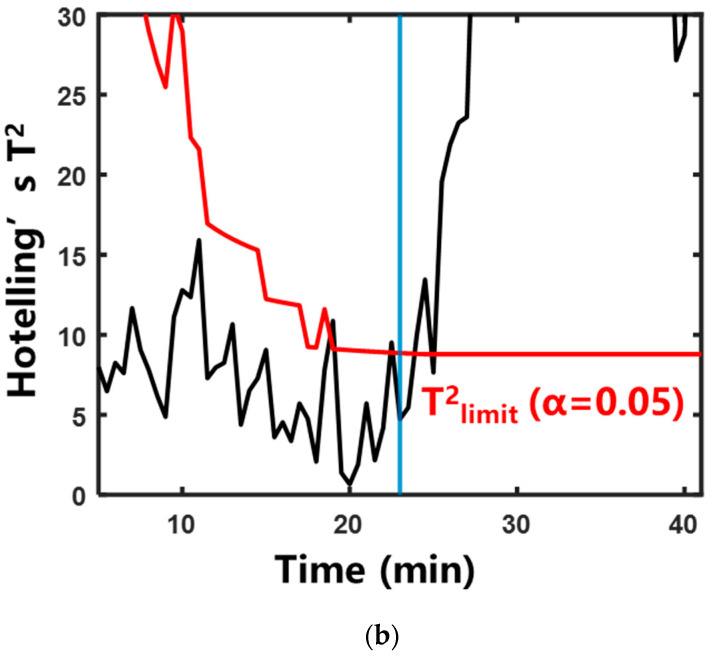
Evaluation of saturated boiling onset timing determined by BoilStart under varying extraction system mass. The blue vertical line indicates that the system entered saturated boiling: (**a**) 120 g; (**b**) 140 g.

Rehmanniae Radix Preparata was used as the object of water extraction. The study included three experimental groups using extraction system masses of 100 g, 120 g, and 140 g. The corresponding water additions were 1200 mL, 1440 mL, and 1680 mL, respectively. Figure S1 shows the variation trends of temperature and AE signal at each phase of the boiling stage. The result of 100 g is shown in Figure 6a. With the increase in the mass of the whole extraction system, the boiling process was slowed down. The result of BoilStart’s application at 100 g is shown in Figure 9a. The rest of the experimental results are shown in Figure 10 and Table 2. The absolute errors of the BoilStart’s judgment results were 1.8 ± 0.6, 1.7 ± 1.0, and 2.0 ± 0.5 min. This indicated that BoilStart had a satisfactory capability to judge the boiling in the system of different masses. Relative to the temperature method, BoilStart cut the increasing-temperature time by 22.2 ± 5.2%, 26.1 ± 10.7%, and 26.9 ± 3.1%. This showed that BoilStart can enhance efficiency.

#### 2.3.2. Heating Medium Temperature

**Figure 11:**
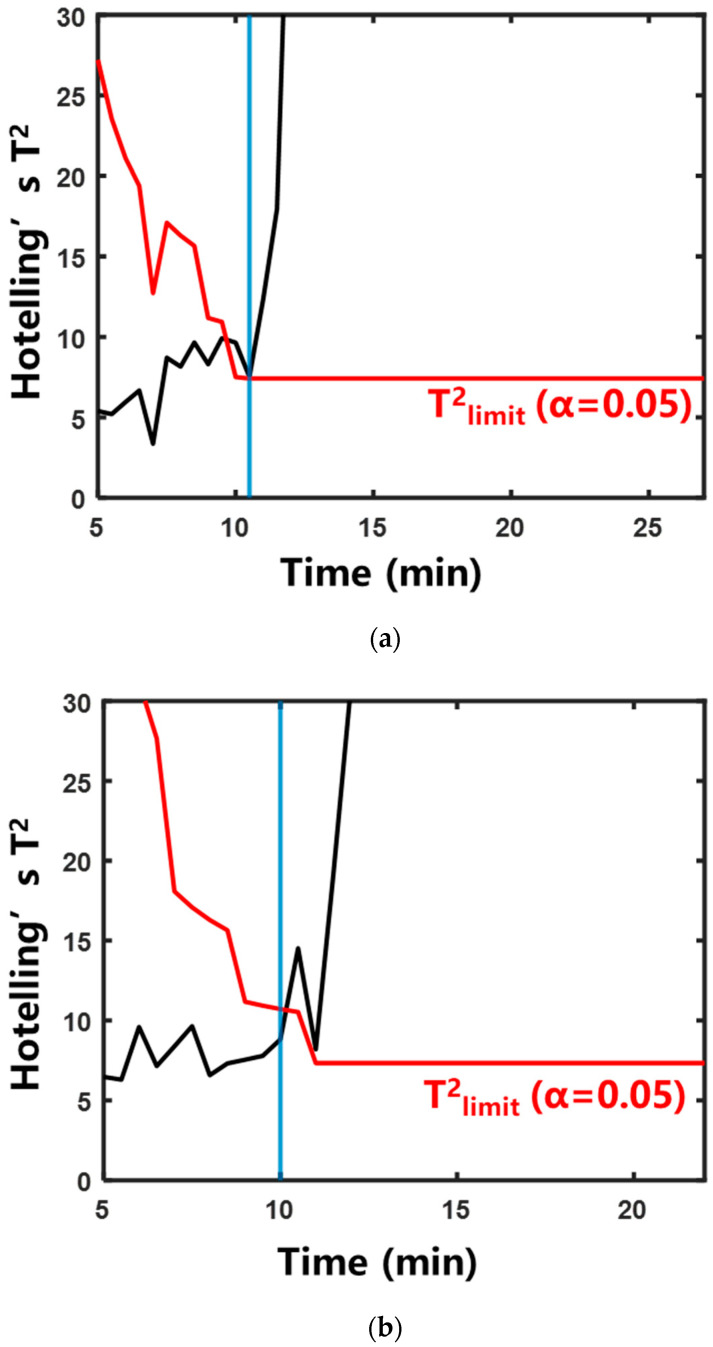
Evaluation of saturated boiling onset timing determined by BoilStart under varying heating medium temperatures. The blue vertical line indicates that the system entered saturated boiling: (**a**) 130 °C; (**b**) 135 °C.

Rehmanniae Radix Preparata was used as the object of water extraction. Three experimental groups were conducted, with heating medium temperatures set to 125 °C, 130 °C, and 135 °C, respectively. Figure S2 shows the variation curves of temperature and AE signal at each phase of the boiling stage under different heating medium temperatures. The result for 125 °C is shown in Figure 6a. With the increase in heating medium temperature, the boiling process was accelerated. The result for 125 °C is shown in Figure 9a. The rest of the experimental results are shown in Figure 11 and Table 2. The absolute errors were 1.8 ± 0.5, 2.0 ± 1.7, and 1.5 ± 1.3 min. It showed that the method can realize the accurate judgment of the boiling moment at different heating medium temperatures. Against the temperature method, BoilStart shortened increasing-temperature time by 22.5 ± 5.2%, 30.1 ± 16.4%, and 27.6 ± 11.0%. This demonstrated BoilStart’s efficiency gains.

### 2.4. Application of BoilStart in Dabuyin Wan

**Figure 12:**
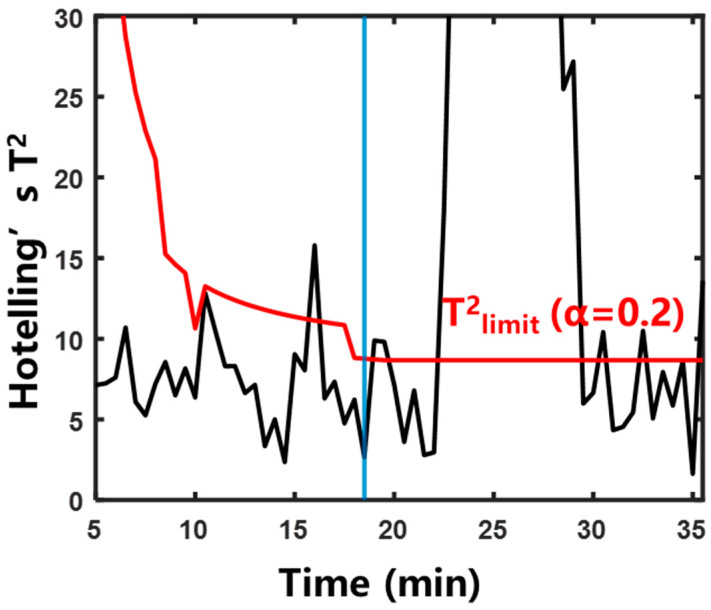
Application of BoilStart in the extraction process of Dabuyin Wan. (The blue vertical line indicates that the system entered saturated boiling.)

In the production of Dabuyin Wan, Rehmanniae Radix Preparata, Phellodendri Chinensis Cortex, and Anemarrhenae Rhizoma were decocted together. To verify the applicability, the laboratory tests were conducted and reproduced the industrial extraction parameters. BoilStart was applied to monitor the boiling process of the combined decoction in Dabuyin Wan. A total of 100 g of Rehmanniae Radix Preparata, Phellodendri Chinensis Cortex, and Anemarrhenae Rhizoma were added with 12 times of water. The temperature of the heating medium was set to 125 °C. The AE and temperature signals were collected during the increasing-temperature process. As shown in Figure S3, the extraction system of Dabuyin Wan entered saturated boiling at 19.3 ± 08 min, at which the power spectral density increased rapidly. Figure 12 shows that BoilStart judged boiling to be at 20.8 ± 1.2 min, indicating the good accuracy of the method. Compared with the boiling criterion of 100 °C, BoilStart can recognize the saturated boiling stage of 8.3 ± 0.3 min earlier. The increasing-temperature time was shortened by about 29%, which improved production efficiency and saved energy. In addition, the electricity meter was used to measure the electricity consumption of this process. The carbon emissions were calculated based on the 2022 Electricity Carbon Dioxide Emission Factor of Zhejiang Province published by the Ministry of Ecology and Environment of the People’s Republic of China [36]. The calculation formula is shown in Equation (1). The results showed that using BoilStart could reduce the electricity consumption during the increasing-temperature time by approximately 26%.

(1) CE=EF×EC

where CE is the carbon emissions; EF is the electricity carbon dioxide emission factor (0.5153 kgCO_2_/kWh); and EF is the electricity consumption measured by the electricity meter.

## 3. Discussion

### 3.1. Advantages of BoilStart

At the present stage, the boiling state recognition of the industrial extraction process relies on manual experience or the built-in temperature sensor of the extraction tank. This results in a lag in the determination of the boiling moment. Compared with the current industrial methods, the advantages of BoilStart are as follows in three points: First, as shown in Figure 2, Figure 4, Figure 6 and Figures S1–S3, the changes in AE signal at different boiling stages were more obvious. When the system entered saturated boiling, the AE signal intensity increased significantly, while the temperature rise rate was very low, making it difficult to accurately distinguish subcooled boiling and saturated boiling. Second, as shown in Table 2, BoilStart was adaptive under different extraction conditions. It can update the threshold in real time through iterative modeling. Under different extraction conditions, BoilStart’s boiling judgment error was within 1.5 to 2.0 min. The temperatures at the boiling moment may vary under different extraction conditions and medicinal material systems. It ranged from 88.1 °C to 92.8 °C in this study. BoilStart could adapt to these differences. Third, BoilStart was beneficial for efficiency improvement and energy conservation. BoilStart could promptly detect whether the extraction system has entered the discrete saturated boiling state. Temperature-based monitoring often suffered from lag in identifying the boiling onset. This led to unnecessary heating energy consumption and time consumption. According to Table 2, under the same extraction conditions, using BoilStart can save approximately 22% to 36% of increasing-temperature time compared to using temperature.

### 3.2. Influence of Herb Characteristics on AE Signals

**Figure 13:**
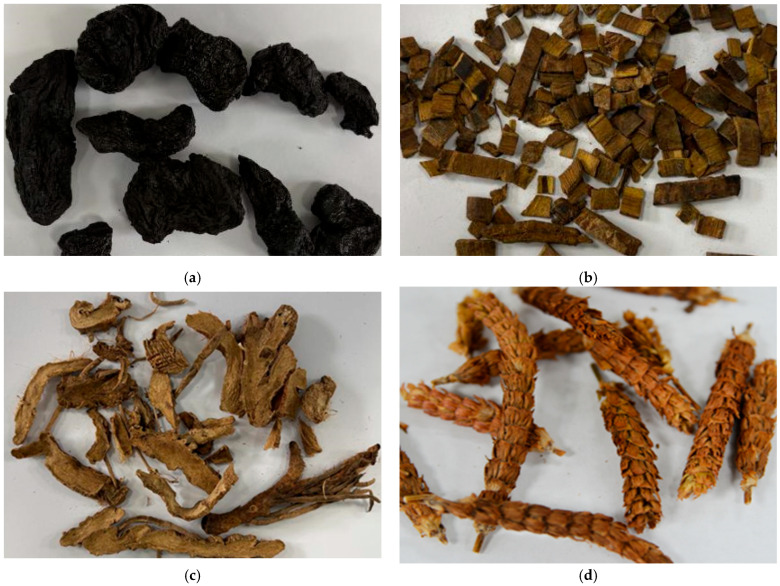
Morphology of herbs: (**a**) Rehmanniae Radix Preparata; (**b**) Phellodendri Chinensis Cortex; (**c**) Anemarrhenae Rhizoma; (**d**) Prunellae Spica.

The herbs used in this paper are shown in Figure 13. Rehmanniae Radix Preparata is the tuberous root, Phellodendri Chinensis Cortex is the bark, Anemarrhenae Rhizoma is the rhizome, and Prunellae Spica is the spica. The different morphology of the herbs may change the bubble behavior and the corresponding acoustic characteristics. The time to reach saturated boiling in the Prunellae Spica system was earlier than that of the other herb systems. This may be attributed to the fact that the loose and porous surface structure of Prunellae Spica provided more bubble nucleation sites and accelerated the boiling process [37]. In addition, the power spectral density of the Prunellae Spica system went directly into up and down fluctuations after a rapid rise, and it is hypothesized that its morphology influenced the bubble aggregation [38].

**Figure 14:**
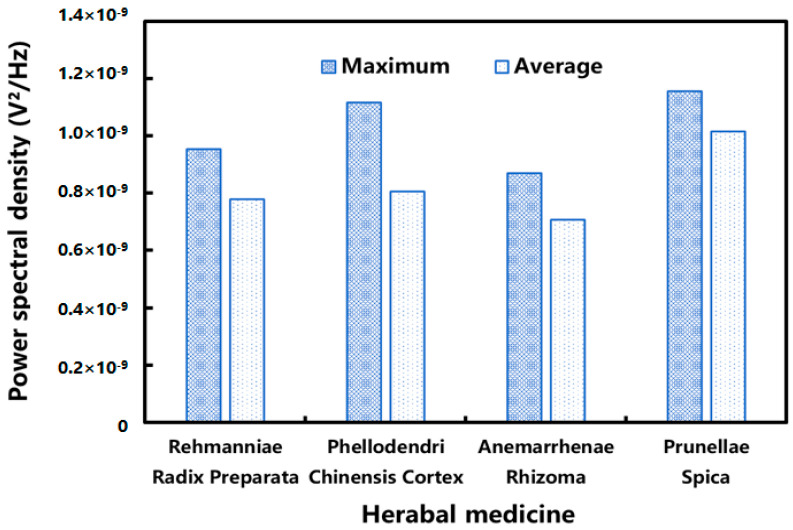
Maximum and average values of power spectral density at “signature frequencies” during the aqueous extraction of different herbs. (Both the axes and the color bars were linear. The power spectral density was presented in linear units (V^2^/Hz) and displayed in scientific notation.)

In addition to the morphology of the herbs themselves, the components in the herbal extracts may also affect the acoustic characteristics during the boiling process. The maximum and mean values of the power spectral density of the discrete saturated boiling stage were calculated. As shown in Figure 14, the overall value of the power spectral density of the Anemarrhenae Rhizoma system was relatively small. The literature reported that the Anemarrhenae Rhizoma extract contained more saponin components [39]. Since saponins reduced the surface tension of the aqueous solution [40], it is speculated that the presence of saponins made the bubbles more stable. The energy intensity of the AE signals generated by bubble rupture was reduced accordingly. After the onset of saturated boiling, the 75–100 kHz band showed a pronounced increase in power spectral density in all four extraction systems. Both the signature frequencies and the power spectral density magnitude differed across systems. These observations suggest correlation of AE features with herbal compositions or forms.

### 3.3. Hotelling’s T^2^ for AE Monitoring

In this study, the AE signal related to boiling was concentrated in the 75–100 kHz band. Its spectral features varied with extraction conditions. To retain the relevant information while ensuring robustness, the power spectral density within the selected frequency band was compressed using PCA, and the process was monitored through Hotelling’s T^2^ and SPE. Hotelling’s T^2^ measured deviations were within the principal component subspace. SPE measured deviations in the residual subspace. When the boiling state changed, Hotelling’s T^2^ and SPE deviated significantly from the baseline. Hotelling’s T^2^ exhibited smaller trigger time errors, suggesting that it is suitable for online monitoring.

Machine-learning classifiers and neural networks typically require larger labeled datasets [41,42,43] and higher computational budgets [44,45,46]. In contrast, Hotelling’s T^2^ is efficient and lightweight. It is also easier to audit and maintain. With only a small number of nominal batches, it can establish models and control limits. However, compared with the commonly used method in the industry of determining boiling based on temperature, workers may feel the present method more difficult to understand.

### 3.4. Limitations and Future Work

This study did not apply BoilStart to the factory site. The noise impact at the production site has not been investigated yet. According to the literature [47], the noise of the steam pipeline and valve was mainly concentrated within 0–2 kHz, whereas our AE band was 75–100 kHz. These above bands did not overlap. However, there may still be other interfering sound sources in industrial sites. In future studies, we will try to use BoilStart in industrial sites to observe the application effect.

In this study, the robustness of AE-based BoilStart at different heating medium temperatures and extraction system masses was investigated. However, we did not quantitatively study bubble size or its distribution. Prior studies [21,48] have shown that the variation in heat flux altered bubble generation frequency and size distribution, and concurrently changed AE characteristics. Therefore, it is necessary to study the relationship between bubble size and AE signal in the future. For instance, high-speed cameras can be used to collect information on bubble size and nucleation frequency. Machine-learning methods can be adopted to establish the relationship between bubbles and acoustic emission signals, etc.

The medicinal materials of traditional Chinese medicine are generally processed through steps such as cleaning and cutting to become decoction pieces. In the industry, the decoction pieces are mostly directly extracted without additional grinding. To ensure consistency with practical conditions, our experiments used decoction pieces without grinding. However, the particle-size distribution may affect AE features. Therefore, further research is still needed on the influence of the particle-size distribution of decoction pieces on the results of BoilStart.

## 4. Materials and Methods

### 4.1. Selection of Experimental Medicinal Materials

Rehmanniae Radix Praeparata, Phellodendri Chinensis Cortex, Anemarrhenae Rhizoma, and Prunellae Spica were selected as representative materials for two reasons. First, these herbs represent four different medicinal parts: tuberous root, bark, rhizome, and spica. Such differences may influence the density of nucleation sites, bubble generation and coalescence behavior, and consequently AE spectral signatures [37,38]. This diversity demonstrated the cross-part applicability of BoilStart. Second, Anemarrhenae Rhizoma contains high levels of saponins [39], which have been reported to reduce the surface tension of aqueous media [40]. Therefore, the bubble behavior in its extraction system may differ, in turn affecting AE intensity and spectral distribution. The batch numbers and sources of the medicinal materials used in this experiment are as follows: Rehmanniae Radix Preparata (JP20231030-4), Phellodendri Chinensis Cortex (JP20230112-4), Anemarrhenae Rhizoma (JP20230627-1) were provided by Hangzhou Hu Qing Yu Tang Pharmaceutical Co., Ltd. (Hangzhou, China). Prunellae Spica (2022112306) was provided by Chiatai Qingchunbao Pharmaceutical Co., Ltd. (Hangzhou, China).

### 4.2. Experimental Setup

**Figure 15:**
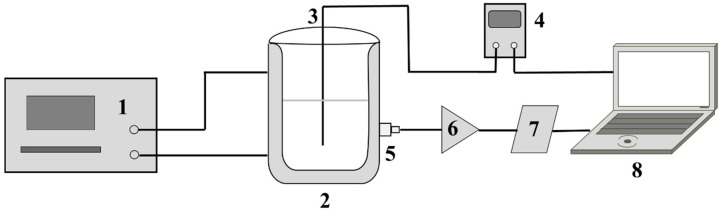
AE signal acquisition setup for herbal medicine extraction. (1) High-temperature circulator, (2) glass-jacketed reactor, (3) K-type thermocouple probe, (4) thermocouple temperature measuring instrument, (5) AE sensor, (6) AE signal conditioner, (7) data acquisition card, (8) workstation.

The AE signal acquisition setup for the herbal extraction process is shown in Figure 15. A glass-jacketed reactor with an effective volume of 2000 mL was used as the extraction vessel. It was heated by a high-temperature circulator (HMGX-2005, Yancheng Hailan Instrument Co., Ltd., Yancheng, China). The system temperature was measured by a K-type thermocouple probe (TB601-1A, Suzhou Zoff Electronics Co., Ltd., Suzhou, China) and a thermocouple temperature measuring instrument (TA612C, Suzhou Zoff Electronics Co., Ltd., Suzhou, China). AE signal acquisition system consisted of an AE sensor (8152C0, Kistler Group, Winterthur, Switzerland), an AE signal conditioner (5125C, Kistler Group, Winterthur, Switzerland), and a data acquisition card (NI9223, National Instruments, Austin, TX, USA). The acoustic and temperature data were recorded and saved in real time by the workstation. The magnification of the signal conditioner was set to 1000. The sampling rate was 1 MHz. The signal acquisition period was 30 s. The collection duration was 10 s. An electricity meter (DL333501, Deli Group Co., Ltd., Ningbo, China) was used to measure electricity consumption.

During the experiment, when the heating medium temperature reached the set value, pure water or extracted herbs and the corresponding multiples of water were added. Then, AE signal and temperature signal were acquired every 30 s. The signal collection was stopped 5 min after the system began to boil. The acquired signals were subjected to spectrum analysis and feature extraction.

### 4.3. Performance Evaluation of BoilStart

To assess methodological applicability, BoilStart was applied to the aqueous extraction processes of four herbal materials. Each experiment was repeated three times. Detailed extraction parameters are provided in Table 3. The phenomenon of bubbles rising to the liquid surface after detachment from the vessel wall was regarded as the indication that the system entered saturated boiling. The accuracy of BoilStart’s boiling judgment was assessed using the absolute error time (AET), defined by Equation (2). Compared with conventional temperature monitoring, BoilStart’s effects on improving production efficiency and reducing energy consumption were examined. The efficiency gain of BoilStart was quantified by the time shortening ratio ($\delta_{time}$), defined by Equation (3):

(2) $$AET=|t_{ref}-t_{AE}|$$

where $t_{ref}$ is the boiling moment determined based on the bubble behavior, and $t_{AE}$ is the boiling moment determined by BoilStart.

(3) $$\delta_{time}=\frac{t_{temp}-t_{AE}}{t_{temp}}\times 100\%$$

where $t_{temp}$ is the boiling moment determined by temperature sensor. The boiling standard was adopted at 100 °C, which is commonly used in the industry.

The robustness of BoilStart in boiling detection and energy efficiency improvement under different conditions was evaluated by calculating AET and $\delta_{time}$. Rehmanniae Radix Praeparata was used as an experimental material. Three mass levels were set for the extraction system: 100 g, 120 g, and 140 g. For the heating medium temperature, three levels were set: 125 °C, 130 °C, and 135 °C. Each experiment was repeated three times. Detailed extraction parameters are provided in Table 3. The pH was measured on samples taken at the boiling point.

### 4.4. Data Analysis

#### 4.4.1. Data Pre-Processing

In AE signal analysis, the time domain signal was converted to the frequency domain through spectral analysis. In this study, the power spectral density estimation method was used for the spectral analysis of time domain signals. Firstly, each raw signal segment obtained from acquisition was divided into 80 segments. Each signal segment was centered to remove the DC bias. The power spectrum was calculated using the periodogram function in the built-in signal processing toolkit of MATLAB 2022a. For the AE signal $X_{n}$ with sampling frequency $f_{s}$, the power spectral density estimation method based on the discrete Fourier variation is shown in Equation (4) [49]:

(4) $$\hat{P}\left(f\right)=\frac{1}{f_{s}N}{\left|\sum_{n=0}^{N-1}{h_{n}x}_{n}e^{-j2\pi fn/f_{s}}\right|}^{2}$$

where $\hat{P}\left(f\right)$ is the power spectral density estimate at frequency f, N is the total number of signal points, $h_{n}$ is the window function, and $x_{n}$ is the signal value of the discrete signal at time point *n*. The window function was set as Hann window to suppress the spectral leakage; $f_{s}$ was 1 MHz, and N was 125,000. The one-sided spectrum was calculated to retain the effective frequency information from 0 to 500 kHz. The power spectrum of the original signal was estimated by averaging all the segmented power spectra. To further reduce the effect of random noise, the spectrum was uniformly segmented into 500 sub-bands. Each sub-band had a bandwidth of 1 kHz. The dimensionality-reduced spectrum was used to construct the AE spectrum dataset for the extraction process.

#### 4.4.2. Multivariate Statistical Analysis

After obtaining the AE spectrum dataset of the extraction process, different boiling stages were divided based on different bubble behaviors. “Signature frequency” was selected, whose power spectral density variation trend was consistent with boiling stage change and whose power spectral density signal strength was relatively large. However, “signature frequencies” of AE signals were different in different herbal extraction systems. The manual screening was inefficient. Therefore, multivariate statistical analysis was introduced into this study. The AE signals in the frequency band were compressed into a few statistics by principal component analysis (PCA). In this paper, Hotelling’s T^2^ and SPE statistics were compared. Hotelling’s T^2^ statistic was obtained by co-accumulating the normalized scores of all principal components. For the data matrix X(N×K), Hotelling’s T^2^ statistic of the *n*-th sampling point $X_{n}(1\times K)$ is calculated as shown in Equation (5). It can be used to determine whether an observation significantly deviated from the center of other observations.

(5) $$T_{n}^{2}=(t_{n}-\bar{t})\lambda^{-1}{\left(t_{n}-\bar{t}\right)}^{T}$$

where $t_{n}(1\times A)$ is the vector consisting of the scores of *A* principal components of the sampling point $X_{n}$, $\bar{t}(1\times A)$ is the vector consisting of the mean values of the scores of each principal component over all *N* sampling points, and λ(1×A) is the diagonal matrix consisting of the corresponding eigenvalues of *A* principal components. Under the assumption that *X* obeys a multivariate normal distribution, the control limit of Hotelling’s T^2^ statistic is calculated using the *F* distribution as shown in Equation (6):

(6) $$T_{UCL}^{2}=\frac{A(N-1)}{\left(N-A\right)}F_{1-\alpha }(A,N-A)$$

where $F_{1-\alpha }(A,N-A)$ is the upper critical value at significance level *α* of the *F* distribution obeying the degrees of freedom (A,N−A).

The SPE statistic is the sum of squares of the model residuals. It represents the variation in the sampling points not explained by the model. The SPE statistic for the *n*-th sampling point $X_{n}(1\times K)$ is calculated as shown in Equation (7). It is used to measure the difference between the observed values and the predicted values of the PCA model.

(7) $${SPE}_{n}=e_{n}e_{n}^{T}=\sum_{k=1}^{k}{\left(x_{nk}-{\hat{x}}_{nk}\right)}^{2}$$

where $e_{n}$ is the residual term of the sampling point $X_{n}$, $x_{nk}$ is the observed value of the *k*-th variable of the sampling point $X_{n}$, and ${\hat{x}}_{nk}$ is the model predicted value of the *k*-th variable of the sampling point $X_{n}$. Assuming that SPE obeys binomial distribution, when the significance level is α, the calculation of SPE control limits is shown in Equation (8):

(8) $${SPE}_{\alpha }=\theta_{1}{\left[\frac{z_{\alpha }h_{0}\sqrt{2\theta_{2}}}{\theta_{1}}+\frac{\theta_{2}h_{0}\left(h_{0}-1\right)}{\theta_{1}^{2}}+1\right]}^{\frac{1}{h_{0}}}$$

where

(9) $$\theta_{i}=\sum_{k=A+1}^{K}\lambda_{k}^{i}(i=1,2,3)$$

(10) $$h_{0}=1-\frac{2\theta_{1}\theta_{3}}{3\theta_{2}^{2}}$$

$Z_{\alpha }$ is the 100 (1 − *α*)% confidence limit of the standard normal distribution, and $\lambda_{k}$ is the *k*-th eigenvalue of the *X* covariance matrix.

To compare the ability of Hotelling’s T^2^ and SPE statistics to distinguish saturated boiling, the extraction process of Rehmanniae Radix Preparat was taken as a research case. A multivariate statistical analysis model was established by selecting the power spectra in the 75–100 kHz band in the subcooled boiling stage of each individual batch. Through PCA dimensionality reduction, the principal components with an explanatory variation ability greater than 0.05 were retained. The batch control limits of Hotelling’s T^2^_limit_ (*α* = 0.01) and SPE_limit_ (*α* = 0.01) were established. Hotelling’s T^2^ and SPE statistics of each time point after the start of the extraction were calculated. The selected statistics were those whose moments of exceeding the control limits were closer to the moments when the system entered saturated boiling.

#### 4.4.3. BoilStart

**Figure 16:**
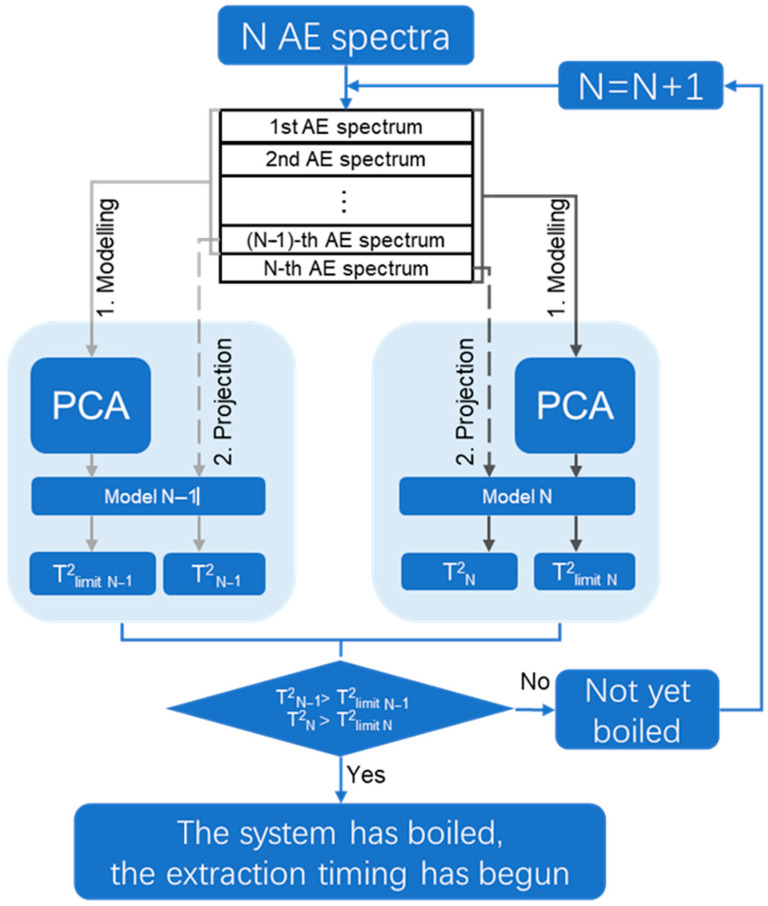
Calculation flow diagram of BoilStart.

In order to realize the real-time accurate judgment of the starting point of boiling, this study proposed a self-adaptive decision-making boiling judgment method named BoilStart, based on iterative multivariate statistical analysis. As shown in Figure 16, real-time modeling and monitoring were carried out by using AE signals acquired from each individual batch. The specific steps were as follows: iterative modeling was carried out starting from N = 10. After acquiring N spectra, PCA was performed on the 1st to the (N − 1)-th AE spectra. The batch control limit T^2^_limit N−1_ was established. The (N − 1)-th spectrum was projected onto the PCA model in real time and its Hotelling’s T^2^ statistic T^2^_N−1_ was calculated. T^2^_N−1_ was compared with T^2^_limit N−1_. Subsequently, PCA was carried out on the 1st to N-th AE spectra. The batch control limit T^2^_limit N_ was established. The N-th spectrum was projected onto the updated model, and T^2^_N_ was computed. A comparison between T^2^_N_ and T^2^_limit N_ was conducted. These steps were repeated until both conditions T^2^_N−1_ > T^2^_limit N−1_ and T^2^_N_ > T^2^_limit N_ were satisfied. Once met, the system was considered to have boiled. A detection signal was triggered by the system.

## 5. Conclusions

In this study, an online AE signal acquisition system was first established. The boiling process was divided into two stages, subcooled boiling and saturated boiling, according to the change in bubble behavior. Spectrogram analysis revealed distinct AE signal peaks within the 75–100 kHz frequency band. Then, the spectral characteristics of the AE signal under different extraction systems and its feasibility of monitoring the boiling state were investigated. The results showed that the AE signal could effectively detect the saturated boiling stage, but the “signature frequency” varied according to herb species. In order to improve the convenience and flexibility of the method, the multivariate statistical analysis method was adopted in this study. Hotelling’s T^2^ statistic was screened out to reflect the boiling state. On this basis, a self-adaptive decision-making method, named BoilStart, was developed using iterative multivariate statistical analysis. BoilStart can realize the online monitoring of the boiling states of different herb systems without a priori model library. The absolute errors between the boiling judgment time and the real value were 1.8 ± 0.6 min, 2.0 ± 0.5 min, 1.8 ± 0.8 min, and 1.8 ± 0.6 min. This showed that the judgment results were accurate and reliable. The robustness of the method under different extraction conditions was evaluated by changing the conditions of extraction quality and heating temperature. The results showed that BoilStart can still maintain a strong boiling judgment ability under different extraction conditions. At heating medium temperatures of 125–135 °C, the absolute errors of BoilStart ranged from 1.5 min to 2.0 min. The increasing-temperature time was reduced by about 22.2–30.1%. At extraction herbal masses of 100–140 g, BoilStart yielded absolute errors of 1.7–2.0 min. The increasing-temperature time was reduced by about 22.5–26.9%. Finally, BoilStart was applied to the extraction process of Dabuyin Wan. BoilStart can more accurately and timely determine the boiling situation of the system compared with the traditional temperature judgment standard. The increasing-temperature time of extraction was shortened by about 29%. The electricity consumption during the increasing-temperature time was reduced by approximately 26%. In summary, BoilStart provided a novel and effective solution for online monitoring of the boiling status of the herbal extraction process. It contributed to shortening unnecessary increasing-temperature time and lowering the energy consumption in production, offering technical support for the intelligent manufacturing transformation in the herbal medicine industry. BoilStart has not been validated under on-site factory, the effects of bubble size, bubble distribution, and herb particle-size distribution were not investigated and will be explored in future studies.

## Figures and Tables

**Table 1:** Boiling moments of different standards during the water extraction process of different herbal medicine (n = 3).

Extraction Herbal	Time When Temperature Reached 100 °C (min)	Entered Saturated Boiling		Hotelling’s T^2^ (min)	SPE (min)
		Time (min)	Temperature (°C)		
Rehmanniae Radix Preparata	27.7 ± 0.8	19.7 ± 0.6	92.5 ± 0.5	20.5 ± 0.5	20.5 ± 0.5
Phellodendri Chinensis Cortex	27.2 ± 1.6	18.8 ± 0.3	91.9 ± 0.8	20.5 ± 0.9	19.7 ± 0.3
Anemarrhenae Rhizoma	25.3 ± 1.3	18.0 ± 0.5	92.8 ± 2.2	22.2 ± 2.8	16.0 ± 6.7
Prunellae Spica	25.8 ± 1.0	15.8 ± 0.3	88.5 ± 2.7	16.8 ± 0.3	16.8 ± 0.6

**Table 2:** Evaluation of saturated boiling onset timing determined by BoilStart (n = 3).

Extraction Herbal	Herb Mass (g)	Heating Medium Temperature (°C)	Time When Temperature Reached 100 °C (min)	Entered Saturated Boiling		Boiling Time Determined with BoilStart (min)	AET (min)	$\delta_{time}$ (%)
				Time (min)	Temperature (°C)			
Rehmanniae Radix Preparata	100	125	27.7 ± 0.8	19.7 ± 0.6	92.5 ± 0.5	21.5 ± 1.0	1.8 ± 0.6	22.2 ± 5.2
	120	125	30.0 ± 2.2	20.7 ± 0.3	92.2 ± 2.3	22.0 ± 1.7	1.7 ± 1.0	26.1 ± 10.7
	140	125	34.7 ± 1.0	23.3 ± 0.3	91.2 ± 2.1	25.3 ± 0.8	2.0 ± 0.5	26.9 ± 3.1
	100	130	19.7 ± 0.8	11.7 ± 1.0	88.1 ± 2.7	13.7 ± 2.8	2.0 ± 1.7	30.1 ± 16.4
	100	135	17.0 ± 0.5	10.8 ± 0.8	87.1 ± 4.2	12.3 ± 2.1	1.5 ± 1.3	27.6 ± 11.0
Phellodendri Chinensis Cortex	100	125	27.2 ± 1.6	18.8 ± 0.3	91.9 ± 0.8	20.8 ± 0.6	2.0 ± 0.5	23.1 ± 5.5
Anemarrhenae Rhizoma	100	125	25.3 ± 1.3	18.0 ± 0.5	92.8 ± 2.2	16.2 ± 1.2	1.8 ± 0.8	36.2 ± 2.7
Prunellae Spica	100	125	25.8 ± 1.0	15.8 ± 0.3	88.5 ± 2.7	17.7 ± 0.8	1.8 ± 0.6	31.6 ± 0.3
Dabuyin Wan	100	125	29.2 ± 1.0	19.3 ± 0.8	91.3 ± 1.2	20.8 ± 1.2	1.5 ± 0.5	28.6 ± 1.6

**Table 3:** Extract specific process parameters (n = 3).

Extracted Herbs	Herb Mass (g)	Heating Medium Temperature (°C)	pH
Rehmanniae Radix Preparata	100.0; 100.1; 100.0	125.0	4.776 ± 0.045
	120.0; 120.2; 120.1	125.0	4.659 ± 0.070
	140.1; 140.1; 140.0	125.0	4.721 ± 0.107
	100.2; 100.1; 100.1	130.0	4.605 ± 0.043
	100.0; 100.0; 100.1	135.0	4.580 ± 0.042
Phellodendri Chinensis Cortex	100.0; 100.1; 100.2	125.0	5.042 ± 0.047
Anemarrhenae Rhizoma	100.0; 100.0; 100.2	125.0	5.445 ± 0.094
Prunellae Spica	100.2; 100.2; 100.2	125.0	6.088 ± 0.057

## Data Availability

The original contributions presented in this study are included in the article/Supplementary Materials. Further inquiries can be directed to the corresponding author.

## Supplementary Materials

The following supporting information can be downloaded at: https://www.mdpi.com/article/10.3390/ph18101556/s1, Figure S1: The variation trend of the increasing-temperature curve and the power spectral density of the “signature frequency” under different extraction system masses. (The blue vertical line indicated that the system entered saturated boiling. Both the axes and the color bars were linear. The power spectral density was presented in linear units (V^2^/Hz) and displayed in scientific notation.): (a) 120 g; (b) 140 g. Figure S2. The variation trend of the increasing-temperature curve and the power spectral densi-ty of the “signature frequency” under different heating medium temperatures. (The blue vertical line indicated that the system entered saturated boiling. Both the axes and the color bars were linear. The power spectral density was presented in linear units (V^2^/Hz) and displayed in scientific notation.): (a) 130 °C; (b) 135 °C. Figure S3: The variation trend of the increasing-temperature curve and the power spectral densi-ty during the extraction process of Dabuyin Wan. (The blue vertical line indicated that the system entered saturated boiling. Both the axes and the color bars were linear. The power spectral density was presented in linear units (V^2^/Hz) and displayed in scientific notation.)

## Abbreviations

The following abbreviations are used in this manuscript:

AE	acoustic emission
PCA	principal component analysis
