# Supplementary material for: Energy Conservation and Production Efficiency Enhancement in Herbal Medicine Extraction: Self-Adaptive Decision-Making Boiling Judgment via Acoustic Emission Technology
Source: Pharmaceuticals (Basel). 2025 Oct 16;18(10):1556. doi: 10.3390/ph18101556 (PMC12567174; doi:10.3390/ph18101556)
Supplement: Supplementary file 1 [file pharmaceuticals-18-01556-s001.zip › pharmaceuticals-3890517-supplementary.pdf]

Supplementary Materials

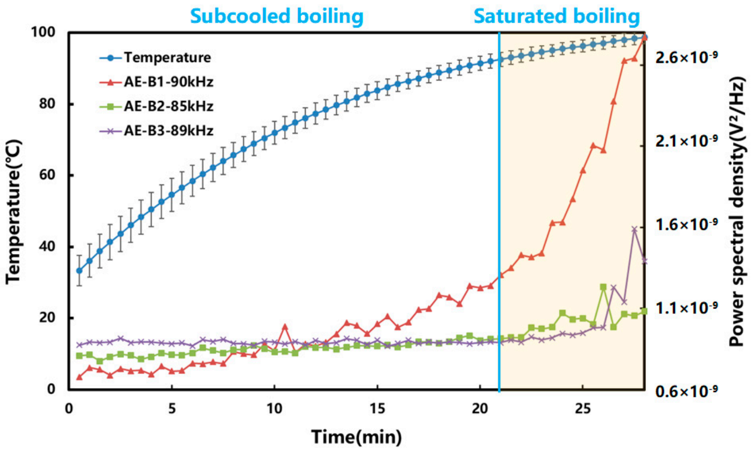

(**a**)

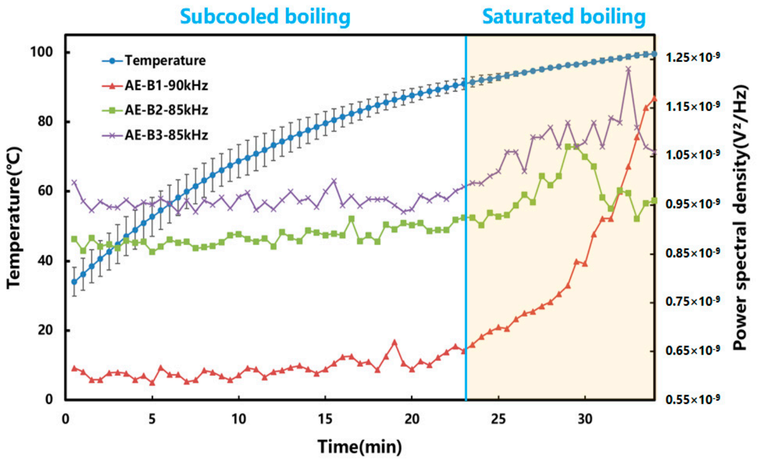

(**b**)

Figure S1. The variation trend of the increasing-temperature curve and the power spectral density of the "signature frequency" under different extraction system masses. (The blue vertical line indicated that the system entered saturated boiling. Both the axes and the color bars were linear. The power spectral density was presented in linear units (V^2/Hz) and displayed in scientific notation.): (**a**) 120 g; (**b**) 140 g.

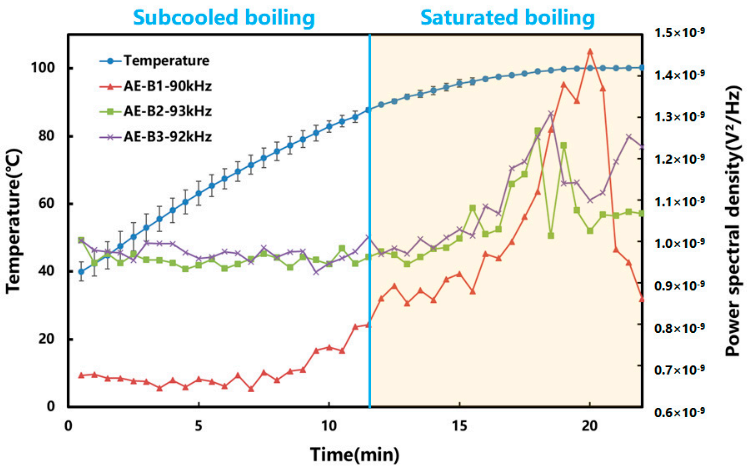

(**a**)

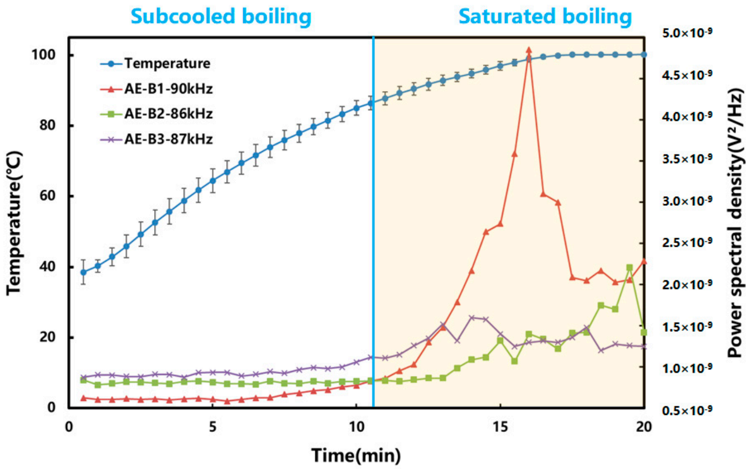

(**b**)

Figure S2. The variation trend of the increasing-temperature curve and the power spectral density of the "signature frequency" under different heating medium temperatures. (The blue vertical line indicated that the system entered saturated boiling. Both the axes and the color bars were linear. The power spectral density was presented in linear units (V^2/Hz) and displayed in scientific notation.): (**a**) 130 °C; (**b**) 135 °C.

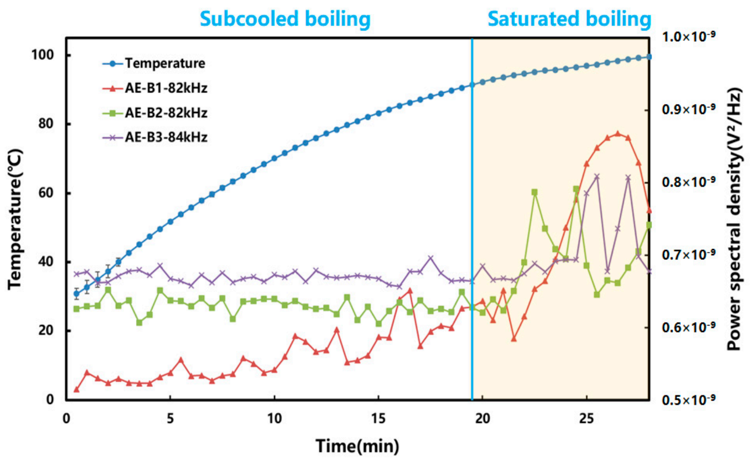

Figure S3. The variation trend of the increasing-temperature curve and the power spectral density during the extraction process of Dabuyin Wan. (The blue vertical line indicated that the system entered saturated boiling. Both the axes and the color bars were linear. The power spectral density was presented in linear units (V^2/Hz) and displayed in scientific notation.)
